# Microarray Analysis Reveals a Potential Role of lncRNA Expression in 3,4-Benzopyrene/Angiotensin II-Activated Macrophage in Abdominal Aortic Aneurysm

**DOI:** 10.1155/2017/9495739

**Published:** 2017-10-18

**Authors:** Yingying Zhou, Jiaoni Wang, Yangjing Xue, Aili Fang, Shaoze Wu, Kaiyu Huang, Luyuan Tao, Jie Wang, Yigen Shen, Jinsheng Wang, Lulu Pan, Lei Li, Kangting Ji

**Affiliations:** ^1^Department of Endocrinology, The Second Affiliated Hospital and Yuying Children's Hospital of Wenzhou Medical University, Wenzhou, Zhejiang, China; ^2^Department of Cardiology, The Second Affiliated Hospital and Yuying Children's Hospital of Wenzhou Medical University, Wenzhou, Zhejiang, China; ^3^Children's Heart Center, The Second Affiliated Hospital and Yuying Children's Hospital, Institute of Cardiovascular Development and Translational Medicine, Wenzhou Medical University, Wenzhou, Zhejiang, China

## Abstract

Abdominal aortic aneurysm (AAA) is a fatal disease, and exposure to 3,4-benzopyrene (Bap) is closely related to the development of AAA. We have found that Bap could impair the biological function of endothelial progenitor cells (EPCs), which are associated with the occurrence of AAA. We have also demonstrated that macrophage activation plays a key role in Bap-induced AAA, but the mechanism is unknown. Here, we used a mouse lncRNA array to investigate the expression signatures of lncRNAs and mRNAs in Bap-activated macrophage. A total of 457 lncRNAs and 219 mRNAs were found to be differentially expressed. The function of differential mRNAs was determined by pathway and Gene Ontology analysis. Eight pathways associated with inflammation were upregulated, and seven pathways including cell apoptosis were downregulated. It was worth noting that AGE-RAGE pathway, which was involved in Bap-induced EPC dysfunction, was significantly upregulated in Bap-activated macrophage and may contribute to AAA formation. Thus, lncRNAs may exert a key role in activated macrophages and intervene the core lncRNAs and may inhibit the occurrence of a series of cascade reactions in the macrophages, which may provide potential targets for AAA caused by smoking.

## 1. Introduction

Abdominal aortic aneurysm, generally defined as the remodeling and expansion of abdominal aorta with an arterial diameter ≥ 30 mm or a 50% increased arterial diameter, is the most common aortic disease on clinic. Because of the high pressure within the aorta, any rupture can quickly lead to death. As reported, an estimated 1% to 3% of men aged 65 to 85 years died of AAA in developed countries [1]. The pathologic mechanism of AAA is still unclear. Increasing evidence has indicated that 3,4-benzopyrene (Bap), an important component of cigarette smoke and automobile exhaust, is one of the leading risk factors of AAA [1, 2]. Our previous study has demonstrated the detrimental effects of Bap on the function of endothelial progenitor cells (EPCs), which are a population of circulating stem cells and closely correlated to endothelial damage and vascular injury [3]. It has been reported that endothelial injury is associated with the occurrence of AAA and the recovery of endothelial integrity correlates with the progression of AAA. Circulating EPCs are reduced, and the function of late EPCs is impaired in AAA patients [4, 5]. Thus, we hypothesize that Bap may play a key role in the development of AAA. Further, we constructed an Angiotensin II- (Ang II-) induced murine AAA model and discovered that Bap could cause pathological change of artery wall similar to AAA and promote the development of AAA [6]. Recently, some studies revealed that macrophage activation and infiltration played a key role in AAA formation [7, 8]. Both the tissue samples of the patients with AAA and the biopsies from AAA model mice clearly exhibited that the infiltration and accumulation of macrophage in the artery wall participate in the whole process of AAA development, from the beginning to expansion and to eventually rupture [9, 10]. We have also confirmed the increment of macrophage infiltration, activation of NF-*κ*B, and expression of MMP-2, MMP-9, and MMP-12 in Ang II/Bap-induced AAA model previously [6]. However, the underlying molecular regulatory mechanism in Bap-induced macrophage activation in AAA, and how to alleviate them, remains to be fully elucidated.

Long noncoding RNA (lncRNA) is defined as transcript noncoding RNA with more than 200 nucleotides. With the wide application of whole length cDNA cloning sequence analysis and genome chip technology, tens or even hundreds of thousands of lncRNAs have been counted. Though once considered as “junk” transcripts of the genome, lncRNA now has been proven to be important in the gene expression and function regulation and actively participates in many pathological processes. Efforts have been made to study the relationship between the lncRNA expression and AAA. Holdt et al. have demonstrated a close association of lncRNA CDKN2BAS (or ANRIL) in chr19q13 with atherosclerosis [11]. Wang et al. indicated that lncRNA-HIF 1 alpha-antisense RNA could interact with mRNA BRG1 in vascular smooth muscle cells in vitro, which may contribute to the pathogenesis of thoracic aortic aneurysms [12]. Recently, Yang et al. identified 3688 lncRNAs and 3007 mRNAs differently expressed between AAA and normal abdominal aortic tissues by microarray. And the lncRNA-mRNA targeting relationships were further identified using computational analysis [13]. However, the functional role of lncRNAs in activated macrophages in AAA is largely unknown.

To systematically study the role of lncRNAs in activated macrophages in AAA, we built gene expression profiles of Bap-activated macrophages and the normal control macrophages using lncRNA and mRNA gene expression microarrays. Nine differentially expressed lncRNAs identified were further confirmed via qRT-PCR. Gene Ontology (GO) and the Kyoto Encyclopedia of Genes and Genomes (KEGG) databases were used to clarify their biological functions.

## 2. Materials and Method

### 2.1. Experimental Animals

Male C57/B6J mice, weighing 35 to 40 g and aging 8 to 10 months (Weitong Lihua Experimental Animal Technology Co. Ltd., Beijing, China), were fed in a specific pathogen-free environment. Mice were divided into four groups, with 12 mice in each. Mice in the control group received a weekly intraperitoneal injection of medium-chain triglycerides (Aabrafaclipophilewl 1349; Gattefosse Co., Lyon, France). Mice in the Ang II group received a daily Ang II (Sigma-Aldrich Co., St. Louis, MO) infusion (0.72 mg/kg) via a subcutaneous osmotic minipump (Alzet Osmotic Pump, Model 2004), in addition to medium-chain triglycerides. Mice in the Bap group received a weekly intraperitoneal Bap (Sigma-Aldrich Co.) injection (10 mg/kg). Mice in the Ang II/Bap group received Ang II (0.72 mg/kg) and Bap (10 mg/kg). Bap was dissolved in medium-chain triglycerides (2 mg/ml). After 6 weeks, mice were euthanized using urethane intraperitoneally.

### 2.2. Aortic Tissue Collection and IF and IHC Staining

After the mice were euthanized, the abdominal and thoracic cavities were exposed, and the aorta was washed with PBS and 4% paraformaldehyde through the left ventricle in turn. The abdominal aorta tissue was carefully separated and fixed in 4% paraformaldehyde for immunohistochemistry. The primary antibodies used in IF and IHC staining were CD68 (ab53444), MMP-9 (ab38898), and TNF-*α* (ab6671). The nuclei were stained with DAPI or DAB. Visualization was performed with a fluorescent microscope.

### 2.3. Macrophage Function

#### 2.3.1. Isolation of Murine Peritoneal Macrophage

Three days before the experiment, 3% sodium thioglycolate was injected intraperitoneally. The mice were sacrificed and placed in 75% ethanol solution 2-3 minutes. After injecting 5 ml HANKS into the abdominal cavity, the abdominal wall was gently squeezed with a hand for more than 20 times and then sucked out the lavage fluid. The suspension was washed with HANKS for 3 times before being resuspended and then was cultured in 37°C, 5% CO_2_ incubator for 3-4 hours. After removal of nonadherent cells with PBS, the adherent cells were then used for the experiment. The mouse macrophage cell line RAW264.7 was purchased from American Type Culture Collection. Cells were cultured in Dulbecco's Modified Eagle's Medium containing 10% fetal bovine serum. When the cells were grown to 90% confluence, subcultivation was performed. DMSO as a solvent for Bap and Ang II, its final concentration in the culture medium did not exceed 0.1% (*v/v*). Cells were randomly divided into 5 groups. In the control group, cells were untreated. In other 4 groups, cells were treated with DMSO or 10 *μ*mol/l Ang II or 20 *μ*mol/l Bap or Ang II plus Bap. Cells were cultured for 2 h and 24 h and then collected for RNA and protein extraction, respectively.

### 2.4. RNA Extraction and Quality Control

Total RNA was extracted from macrophage cells with Trizol reagent (Invitrogen Life Technologies, Carlsbad, CA, USA). After being quantified and qualified, the isolated RNA of high purity was subjected to microarray and quantitative real-time polymerase chain reaction.

### 2.5. Microarray Analysis

An arraystar mouse lncRNA microarray v3.0, which could detect 35,923 lncRNAs and 24,881 protein-coding transcripts, was used to analyze the RNA samples. RNA labeling and array hybridization were conducted as described [14]. The expression levels of lncRNAs and mRNAs were compared between the Bap sample and the DMSO sample. Genes having a fold change > 2 and an adjusted *p* < 0.05 were considered as differentially expressed.

### 2.6. GO and KEGG Pathway Analyses

GO and KEGG pathway analyses were performed by KangChen Bio-tech Co. Ltd. (Shanghai, China), in order to analyze the differentially expressed genes systematically and enrich significant GO terms and KEGG pathways (*p* < 0.05) [15]. The significance of the *p* value was evaluated by the false discovery rate (FDR), and an FDR < 0.05 was recommended.

### 2.7. qRT-PCR Validation Assay

The reliability of the microarray data was validated by comparing the results of microarray and qPCR. Nine randomly selected lncRNAs and their expression levels were further evaluated using the SYBR Green method in a fluorescence real-time PCR (Biosystems, C1000, USA) in triplicate. Primers were designed and synthesized (Table 1). The gene expression levels were normalized to the housekeeping gene *β*-actin. The relative expression of the target genes was calculated as 2^−ΔΔCt^.

### 2.8. Statistical Analysis

Data represented the means ± SD. Student's *t*-test was used for a single comparison of 2 groups. One-way analysis of variance followed by the Bonferroni *t*-test was conducted for a comparison of multiple groups. Differences were considered statistically significant at ^∗^*p* < 0.05 and ^∗∗^*p* < 0.01.

## 3. Results

### 3.1. Bap Exacerbates Macrophage Infiltration, MMP-9, and TNF-*α* Expression in the Aortic Wall of Ang II-Infused Mice

**Figure 1:**
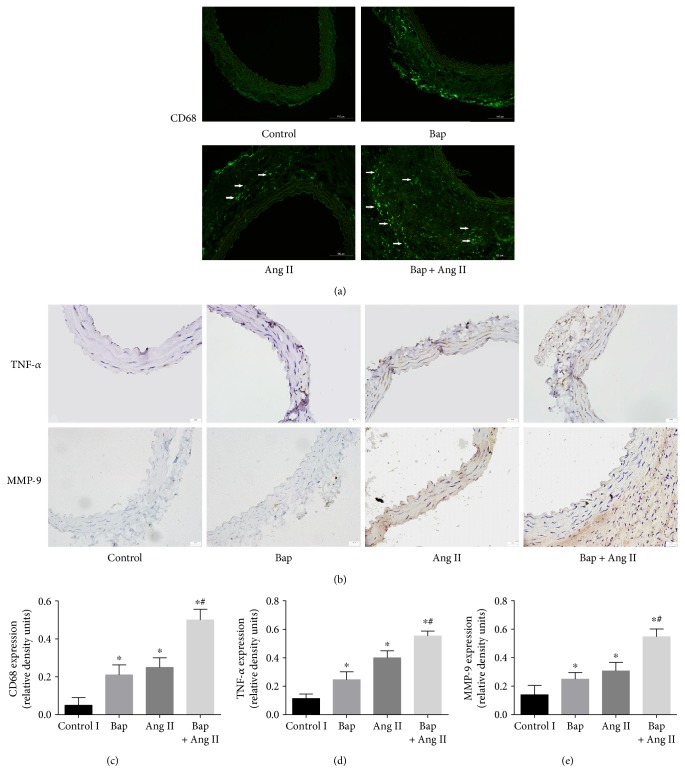
Ang II-induced macrophage infiltration and expression of MMP-9 and TNF-*α* were prominently increased in mice receiving coadministration of Bap. Abdominal aortic tissues were harvested, and transversal sections were prepared. (a) Representative photomicrographs of CD68^+^ cell staining in suprarenal aortic sections: immunoreactivity was visualized using an Alexa Fluor 488 secondary antibody (green). (b) Representative immunohistochemistry staining of TNF-*α* and MMP-9 in the abdominal aorta from control animals and animals treated with Ang II, Bap, or Ang II/Bap. (c–e) For quantitative analysis of the result. ^∗^*p* < 0.05 versus control; ^#^*p* < 0.05 versus Bap or Ang II group.

Immunofluorescence showed prominent macrophage infiltration in abdominal aortic tissues in the Ang II group, which was further promoted by Bap (Figure 1(a)). Because Bap may regulate MMP activity and proinflammatory cytokines, we examined MMP-9 and TNF-*α* expression in abdominal aortic tissue by immunostaining. Ang II infusion increased MMP-9 and TNF-*α* expression when compared with the control group, which was further increased by Bap (Figures 1(b), 1(c), 1(d), and 1(e) for quantitative analysis of the result).

### 3.2. Bap Promotes MMP-9 and TNF-*α* Secretion in Macrophages In Vitro

**Figure 2:**
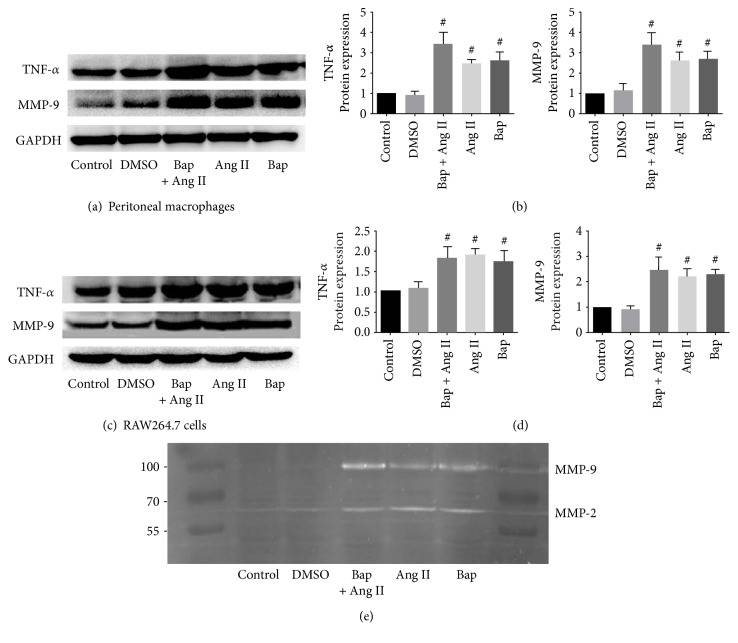
Bap promotes the secretion of TNF-*α* and increases the expression and activity of MMP-9 in macrophages in vitro. (a, b) Representative Western blot analysis of TNF-*α* and MMP-9 in peritoneal macrophages (a) and the quantitative analysis of the result (b). (c, d) Representative Western blot analysis of TNF-*α* and MMP-9 in RAW264.7 cells (c) and the quantitative analysis of the result (d). ^#^*p* < 0.05 versus DMSO group. (e) Gelatin zymography of MMP-9 in RAW264.7 cells.

Chronic inflammation and extracellular matrix degradation have been considered instigating mechanisms underlying AAA. TNF-*α* and MMP-9 are particularly important in this process. Hence, we examined the effect of Bap on the secretion of TNF-*α* and on the expression and activity of MMP-9 in macrophages. As shown in Figure 2, Bap treatment caused significant increment in the amount of MMP-9 and TNF-*α* protein, in both peritoneal macrophages and RAW264.7 cells (*p* < 0.05). Consistently, gelatin zymography demonstrated higher MMP-9 activity in the Ang II group, which was further promoted by Bap (Figure 2(e)).

### 3.3. Microarray Hybridization Data

**Figure 3:**
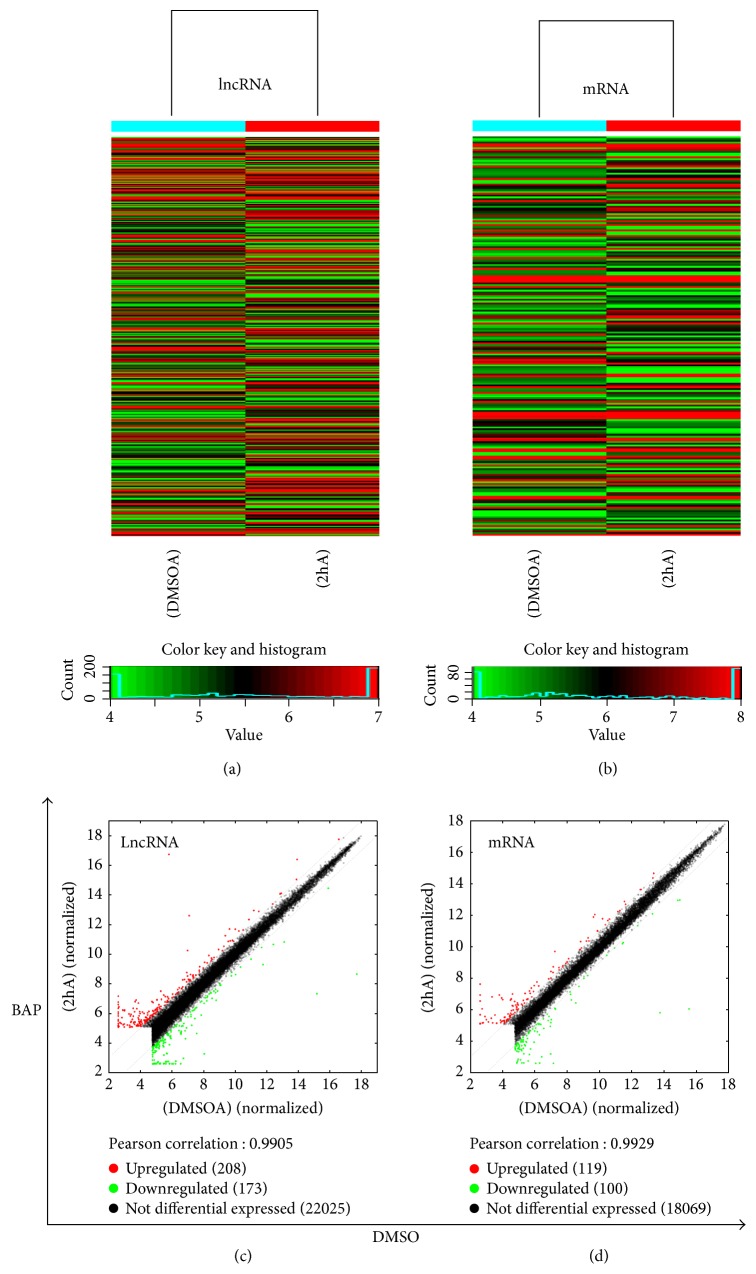
lncRNA and mRNA expression profile comparison between the Bap-activated macrophages and the control group. (a) The hierarchical clustering of all target value lncRNAs and (b) mRNAs. (c) The scatterplot was used for assessing the lncRNA and (d) mRNA expression variation between the Bap-activated cells and the control group. The green lines are fold change lines (the default fold change value given is 2.0). The lncRNAs above the top green line and below the bottom green line indicated >2.0-fold change in expression of lncRNAs between the 2 compared samples. “Red” denotes high relative expression levels, and “blue” denotes low relative expression levels.

Arraystar mouse lncRNA microarray v3.0 is designed for the global profiling of mouse lncRNAs and protein-coding transcripts. The heat map of the hierarchical clustering results was performed to show the distinguishable lncRNA and mRNA expression profiling between the two groups (Figures 3(a) and 3(b)). The results of scatterplot showed that the distribution and expression variation of the log 2 ratios of lncRNAs and mRNAs between the two groups were nearly the same (Figures 3(c) and 3(d)).

### 3.4. Differentially Expressed lncRNAs and mRNAs

We found that 457 detected lncRNAs demonstrated >2-fold differential expression in Bap-activated macrophage when compared to the control group (macrophage cultured with DMSO), with 249 lncRNAs showing upregulation and 208 lncRNAs showing downregulation (Table 2). At the same time, 219 mRNAs displayed beyond a 2-fold differential expression in Bap-induced macrophage, and 119 mRNAs were upregulated while 100 mRNAs were downregulated. The top ten upregulated and top ten downregulated lncRNAs and mRNAs are listed in Table 3.

### 3.5. GO and KEGG Analyses of Differentially Expressed mRNAs

**Figure 4:**
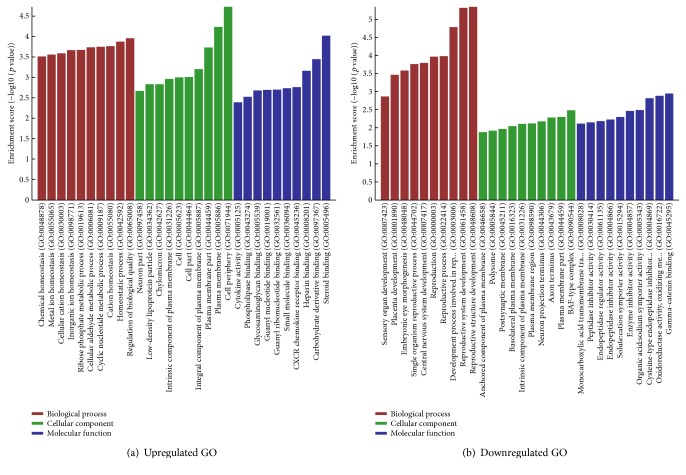
Gene Ontology (GO) analysis of functional classification of the differentially expressed genes. The GO categories cover three domains: biological process, molecular function, and cellular component. (a) The upregulated GO analysis. (b) The downregulated GO analysis. The *p* value denotes the significance of GO term enrichment in the differentially expressed mRNA list. The lower the *p* value is, the more significant the GO term is (*p* value ≤ 0.05 is recommended).

**Figure 5:**
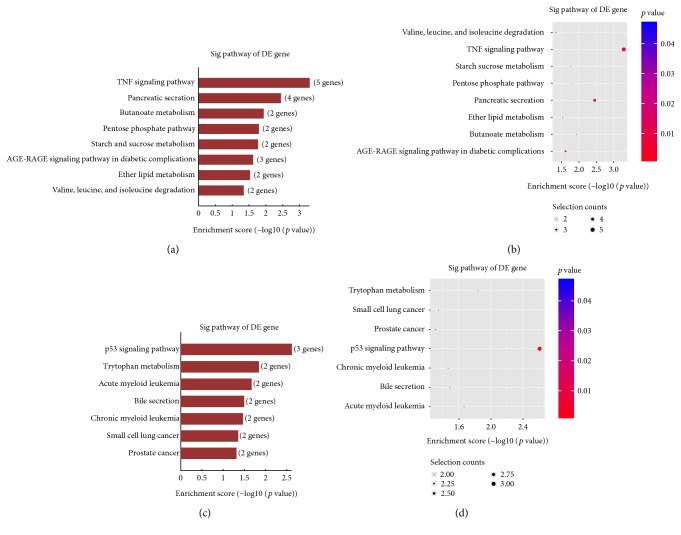
Pathway enrichment analysis. (a, b) The upregulated gene pathway. (c, d) The downregulated gene pathway. The figure shows the top 10 significant pathways of upregulated and downregulated genes. The *p* value (Fisher *p* value) denotes the significance of the pathway correlated to the conditions. The lower the *p* value, the more significant the pathway (the recommended *p* value cut-off is 0.05).

GO analysis, including 3 structured networks: biological processes, cellular components, and molecular function, was applied to analyze the main function of the closest coding genes according to the GO database which provided the key functional classifications for the National Center for Biotechnology Information (NCBI). In our survey, GO analysis revealed the functions of differentially expressed (both upregulated and downregulated) mRNA in abnormally activated macrophages induced by Bap. The most enriched GO terms (top ten) are shown in Figure 4(). Pathway analysis showed that the upregulated mRNAs participated in TNF-*α* signal pathway, AGE-RAGE signaling pathway, and so forth. On the other hand, the involved downregulated mRNAs refer to p53 signaling pathway, tryptophan metabolism, and so forth (Figure 5()).

### 3.6. Validation of the Expression Levels of the lncRNAs Using qRT-PCR

**Figure 6:**
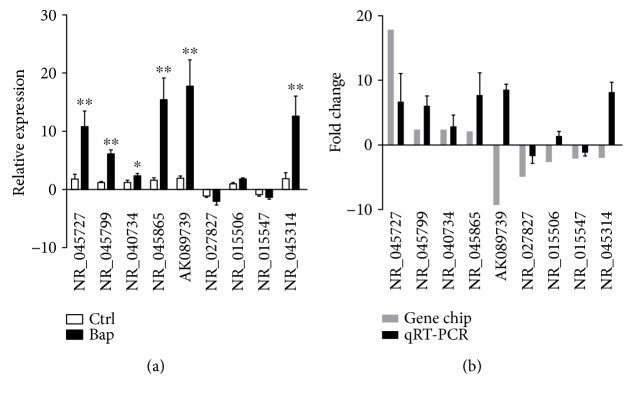
qRT-PCR validation of differential expressions of lncRNAs. (a) Six lncRNAs confirmed by qRT-PCR show to have significant changes between Bap-activated macrophage and the control group. Data are expressed as the mean ± standard deviation (SD) of three independent experiments (^∗^*p* < 0.05 and ^∗∗^*p* < 0.01). (b) qRT-PCR patterns of six lncRNAs are completely consistent with those of microarray data. The *y*-axis represents fold change.

Nine lncRNAs (NR_045727, NR_045799, NR_040734, NR_045865, AK089739, NR_027827, NR_015506, NR_015547, and NR_045314) were selected to validate the microarray consistency by using qPCR. The results demonstrated that NR_045727, NR_045799, NR_040734, and NR_045865 were significantly upregulated in both gene chip and the qRT-PCR (Figure 6).

## 4. Discussion

AAA is a chronic but often fatal vascular disease that is primarily associated with several risk factors including advanced age and smoking [1]. There is lack of effective therapeutic drugs to slow down the development of AAA since the molecular mechanism of AAA is still unclear. Basic research on this disease is urgently needed. Macrophage infiltration into the aortic wall is a hallmark of AAA pathology; thus, targeting vascular inflammation mediated by macrophages may be a potential therapeutic approach for aneurysm pathologies. Our previous study demonstrated that Bap contributed to the pathogenesis of AAA, and indeed, it promoted the formation of AAA in Ang II-treated mice [6]. In the current study, we confirmed that Bap promoted the infiltration of macrophages in the arterial wall of AAA mice in vivo and activates peritoneal macrophages and RAW264.7 cells in vitro. However, how Bap activates macrophages and ultimately promotes the development of AAA is unknown. In order to figure out the probable mechanism, we further carried out a mouse lncRNA profile and identified the potential role of lncRNA expression in activated macrophage in abdominal aortic aneurysm.

lncRNA is a type of noncoding RNAs (ncRNAs). According to the human genome project, the number of total protein-encoding genes in human accounts for <2% of the entire human genome sequence, and 90% of the rest noncoding sequences are transcribed, producing a huge number of ncRNAs [16]. Due to the rapid development of high-throughput RNA sequencing technology, a vast number of new ncRNAs have been discovered. The most well-known ncRNAs are microRNAs (miRNAs), which are ~21–23 nt long and have been proven to play a key role in a variety of biological and pathological processes [17, 18]. Numerous studies have already shown the involvement of microRNAs in AAA development, including miR-195, miR-21, miR-29b, and miR-24 [19–22]. lncRNAs account for 80% of ncRNAs [16]. Previous studies reported that many lncRNAs were involved in cardiovascular disease. Ming et al. found that nicotinamide phosphoribosyltransferase (NAMPT) inhibited EPC senescence through a sirtuin 1 (SIRT1) antisense long noncoding RNA (AS lncRNA)/miR-22/SIRT1 pathway and promoted EPC proliferation and migration [23]. Wu et al. identified lncRNA-p21 as a key regulator of cell proliferation and apoptosis during atherosclerosis through p53 pathway [24]. Viereck and Thum proposed that lncRNAs were involved in the pathological cardiac remodeling, acting as noncoding epigenetic regulators [25]. However, the role of lncRNAs in AAA is less understood. It is a requisite to determine the lncRNA profile about AAA and find key lncRNAs that regulate the pathology of AAA. Therefore, we utilized high-throughput microarray lncRNA screening in this study and discovered differentially expressed lncRNAs in Bap-activated macrophages.

Comparing lncRNA expression in Bap-activated macrophages and the control group to explore features in inflammation of aneurysm disease is a unique approach presented here for the very first time, and the identified pathways added significantly findings to further research in the field. Several significantly changed lncRNAs in our profile were predicted to be closely related to macrophage activation. lncRNA 1700123O21Rik, a 675 nt lncRNA, is located on chromosome 16. Rbfox1 (RNA-binding protein fox-1) is the associated gene of lncRNA 1700123O21Rik. He et al. identified that rare, exonic variants in Rbfox1 had protective effects on BP traits, which could be important in searching new drugs for cardiovascular disease [26]. Hence, Rbfox1 is expressed in multiple tissues that may relate to blood pressure, and the identification of these rare coding variants will facilitate precision medicine in treating cardiovascular disease. In addition, Gao et al. revealed that Rbfox1-dependent RNA splicing, in particular, an isoform switch of MEF2 gene splice variants, was a regulatory circuit in cardiac transcriptional reprogramming, with a significant effect on the pathogenesis of heart failure [27]. We postulated that the expression of Rbfox1 may be involved in inflammation, while the mechanism underlying how this gene influence inflammation needs to be further studied. Another upregulated lncRNA NR_040734, with a FC of 2.4508004, was associated with TMEM30A, which was also known as CDC50A. CDC50A proteins are *β*-subunits for P4-ATPases, which upon heterodimerization form a functional phospholipid translocation complex. Emerging evidence in mouse models and men links mutations in P4-ATPase genes with human disease. Kato et al. indicated that the phospholipid flippase complex of ATP8A1 and CDC50A played a major role in cell migration and suggested that the flippase-mediated translocation of phosphatidylethanolamine at the plasma membrane is involved in the formation of membrane ruffles to promote cell migration [28]. It may be related to macrophage infiltration to the aortic wall. Generally, lncRNAs mainly affect their surrounding associated genes. lncRNA-Angptl2, of which the associated gene was angiopoietin-like 2 (Angptl2), was upregulated in Bap-activated macrophage. Horio et al. found that endothelial cell-derived Angptl2 accelerates vascular inflammation by activating proinflammatory signaling and increasing macrophage infiltration, leading to endothelial dysfunction and atherosclerosis progression [29]. Richardson et al. concluded that Angptl2 positively regulates endothelial colony-forming cell (ECFC) formation in vitro through its effects on migration and in part by activating JNK and increasing MT1-MMP expression [30]. Therefore, we hypothesize that lncRNA-Angptl2 may play a key role in the formation of AAA, through acting on its associated gene Angptl2 and inducing dysfunction of EPCs and macrophages.

Simultaneously, a total of 219 mRNAs, in which119 mRNAs were upregulated and 100 mRNAs were downregulated, were identified as differentially expressed transcripts between the Bap-activated macrophages and the control group. Expression of Crim1 was the most greatly up-altered while DEDD was the most downregulated gene in abnormal activated macrophages induced by Bap. Crim1 has been reported to be necessary for coronary vascular endothelial cell development and homeostasis. Lack of Crim1 in vivo will lead to the malformation of coronary vasculature and the reduced number of endothelial cells [31]. DEDD, identified as death effector domain containing, has been reported to influence mRNA decay and promote cell apoptosis and inhibit cell proliferation [32, 33]. Besides, some of the differentially expressed mRNAs detected in this microarray were associated with the function of stem cells. Among them, IL1R1 and *α*-tocopherol transfer protein have been reported to affect the pathology of AAA. Farhang et al. have demonstrated that repression of TNFR1 and IL1R1 could inhibit NF-*κ*B activation, promote extracellular matrix (ECM) deposition, and allow for maintenance of immunomodulatory properties in inflammatory conditions, which was similar to the pathology of AAA [34]. IL1R1 is a receptor for interleukin beta (IL-1*β*). IL-1β, an important inflammation mediator, has been shown to promote EPC proliferation, migration, and adhesion. Zhang et al. suggested that EPCs could exert self-enhancement effects by interacting with monocytes and that EPCs might also modulate inflammatory reactions by regulating IL-1β expression in monocytes. IL-1*β* has been reported to play a protective role in vascular repair under inflammatory environments [35]. Our chip data showed IL1R1 downregulation in Bap-activated macrophages, which may lead to a low combination of IL-1β to IL1 receptor and result in an increased inflammatory response. SEC14L2, an *α*-tocopherol transfer protein (Ttpa), was expressed in hepatocyte-like cells (HLCs), starting from human mesenchymal stem cells (hMSC) through induced pluripotent stem (iPS) cell reprogramming. Sa-Ngiamsuntorn et al. indicated that, together with the expression of SEC14L2 and the addition of *α*-tocopherol, the expressions of inflammatory cytokines were upregulated during the infection that mimicked the inflammatory process [36].

Studies about the function of lncRNAs are difficult to carry out, for most of the lncRNAs are not determined, and there is no existing database that could be used to find their functional annotations. To solve this problem, we have tried to construct a correlation between mRNA and lncRNA. First, by GO annotation and pathway analysis, we made a systemic analysis for the functions of the differentially expressed mRNAs. TNF-*α* signaling pathway was mostly enriched in the upregulated genes, and p53 signaling pathway was mostly enriched in the downregulated genes in Bap-activated macrophages. Our study and previous other studies found that TNF-*α* signaling pathway played a key role in AAA development [37, 38]. TNF-*α* signaling pathway is also closely related to inflammation and oxidative stress. A recent study demonstrated that TNF-*α* could aggravate inflammatory reactions and oxidative damage in bone marrow-derived mesenchymal stem cells during degenerative bone disease, by upregulating miR-705 and inhibiting FoxO1 [39]. Another high-expression signal pathway involved in the activation of macrophages by Bap was AGE-RAGE signaling pathway, of which the role in the progression of AAA has been reported by Zhang et al. Blocking RAGE in a mouse aneurysm model significantly inhibited the formation of aneurysms and prevented MMP-9 expression in macrophages [40]. This suggests us that lncRNAs acting on the interaction between AGE and RAGE may ultimately lead to novel therapies to treat and prevent AAA progression. Our previous study revealed that RAGE was involved in Bap-induced EPC dysfunction. Bap significantly increased expression of RAGE protein while Astragaloside IV pretreat downregulated the expression of RAGE [6, 41]. It is well known that p53 plays an important role in the pathogenesis of apoptosis [24, 42]. Therefore, we speculate that lncRNAs regulate the activated macrophages in AAA through upregulating TNF-*α* signaling pathway and AGE-RAGE pathway to promote cell inflammatory progression and downregulating p53 signal pathway to inhibit cell apoptosis. Moreover, we need to establish the contact of lncRNA and mRNA in further study. Generally, there are two ways: one way is to use the physical adjacency of the mRNA and lncRNA, and the other is to use the relationship of coexpression pattern among genes.

In summary, we confirmed the function of macrophages in Bap-induced AAA in vivo and in vitro and discovered for the first time a profile of lncRNAs differentially expressed in Bap-activated macrophages in AAA. Our study on lncRNAs has greatly expanded the field of gene research in AAA. Although the mechanisms of the discovered lncRNAs in Bap-activated macrophages remain to be elucidated, we hope that our novel discovery will lead to more studies that will determine its function.

## Figures and Tables

**Table 1:** Primer sequences for lncRNAs.

Sequence name	Gene symbol	Forward (5′-3′)	Reverse (5′-3′)
NR_045727	B230209E15Rik	TCCACTGAACCACCAACCAAA	CCATCTCCGCAAACTGCCTAT
NR_045799	1700123O21Rik	CCTCACTTTAGAGTCCTGGGTA	TTGAAGATTTGCTGTCTGCTG
NR_040734	4930429F24Rik	AGAAGAGGCGTAGGCGTCATA	AGACTTCTGGAGCCGTCAGGT
NR_045865	9230009I02Rik	GGGTTAAGAATCGCATGAGTA	CCAAGAAAACAAGGCAAGAGT
AK089739	AK089739	GGGTCTAACATTTACCAAGATGAAG	TGGAATATCCCCAGAGTCCTA
NR_027827	Chd3os	TCTTTTCCCCAGTATTGCTAC	GTTGACTCCCTGCTTATGATTG
NR_015506	4833418N02Rik	GCACTCAGGATGCTTGGTCTT	CCACTTGCTGCTACTTTATTTTGG
NR_015547	1700009J07Rik	AGGGCATTTTAGTTGGTTCTTACAG	GCAAGCATGGATTCTAGCGTT
NR_045314	9830166K06Rik	TCCCACAGGGTTCAGTTCTCA	GGTCTACATTATTACATCTGGCTCA

**Table 2:** Number of differentially expressed lncRNAs.

lncRNAs	FC 2–5	FC ≥ 5	FC ≥ 10	Total
Up	211	27	11	249
Down	166	33	9	208

**Table 3:** Top 10 upregulated and downregulated lncRNAs and mRNAs in activated macrophage.

Sequence name	Gene symbol	FC
Top 10 upregulated lncRNAs		
NR_040373	Asb17os	1965.641627
ENSMUST00000135495	Ccdc92	45.4580975
uc007cfx.1	AK144617	23.389015
NR_045727	B230209E15Rik	17.9220629
uc007oby.1	AK046721	15.996457
uc007oby.1	AK046721	15.996457
ENSMUST00000173219	Sox2ot	13.8832295
ENSMUST00000145380	Ckmt1	13.4032113
AK083558	AK083558	11.2153303
mouselincRNA1093	mouselincRNA1093	10.2874934
Top 10 upregulated mRNAs		
NM_015800	Crim1	32.4476525
NM_134193	Vmn1r232	20.0515965
NM_023135	Sult1e1	15.5459966
NM_146016	Eml6	11.060142
NM_001105061	Gm9268	10.1305416
NM_001001177	BC051142	8.8110002
NM_182745	1700028K03Rik	8.5059219
NM_030739	Vmn1r58	7.0052416
NM_010157	Esr2	6.3562933
NM_010104	Edn1	6.2573404
Top 10 upregulated lncRNAs		
NR_040395	D430036J16Rik	536.89308
NR_038179	1700042G15Rik	233.8747012
ENSMUST00000120698	Gm13079	27.4479976
ENSMUST00000177106	Gm20614	15.4528389
ENSMUST00000181660	2610017I09Rik	13.5374124
ENSMUST00000176851	Idi1	12.2999829
uc007gkp.1	AK054042	11.1609776
ENSMUST00000140447	1810010H24Rik	11.0132014
ENSMUST00000146208	Gm15270	10.5662387
AK033575	AK033575	9.4937891
Top 10 downregulated mRNAs		
NM_001128609	DEDD	727.5505604
NM_001276250	Cp	245.8931099
NM_001136227	Rtkn	23.4801529
NM_026100	Tctex1d1	13.7970765
NM_029751	Rpl18a	12.9072764
NM_029292	1700008F21Rik	10.9811558
NM_181682	Dsg1b	10.6274348
NM_146689	Olfr1459	7.1381074
NM_173751	Ilvbl	6.4870461
NM_177915	Igsf1	5.9488207
